# Stroke in a Patient With Antiphospholipid Syndrome

**DOI:** 10.7759/cureus.56897

**Published:** 2024-03-25

**Authors:** Mateo Zuluaga-Gomez, Laura Quintero-Gutierrez, Daniel González-Arroyave, Carlos M Ardila

**Affiliations:** 1 Emergency Department, Hospital San Vicente Fundacion, Medellin, COL; 2 Surgery, Universidad Pontificia Bolivariana, Medellin, COL; 3 Basic Sciences, University of Antioquia, Medellin, COL

**Keywords:** emergency medicine, thrombophilias, stroke, antiphospholipid antibody, antiphospholipid syndrome

## Abstract

Antiphospholipid syndrome (APS) is characterized by the occurrence of thrombotic events and/or obstetric complications in the presence of antiphospholipid antibodies. It is considered one of the most common acquired thrombophilias. The presentation of stroke in patients with APS has been described in some studies; however, it is not frequent enough and there is not much information available regarding the indications for pharmacological thrombolysis and the safety of thrombolytic treatment. Likewise, current evidence does not describe contraindications to thrombolytic therapy in cases of this diagnosis, which makes management with fibrinolysis safe in these cases. A clinical case of stroke is presented in which pharmacological thrombolysis is performed with a successful outcome, without complications of angioedema or bleeding. Likewise, concerning the case, the main neurological manifestations associated with APS, especially in its association with stroke, are described.

## Introduction

Antiphospholipid syndrome (APS) is defined by the occurrence of thrombotic events and/or obstetric complications in the presence of antiphospholipid antibodies (APL). It is considered one of the most common acquired thrombophilias. Over the long term, patients with APS have an increased risk of recurrent thrombotic events in both venous and arterial circulation [1]. This syndrome is secondary to the production of antibodies against various cell membrane lipids: anticardiolipin antibodies (immunoglobulin (Ig)G and IgM), anti-beta-2 glycoprotein-1, and lupus anticoagulant [1,2]. Depending on its origin, APS can be primary or secondary. Classic primary APS refers to obstetric complications or arterial and venous thrombotic events. Secondary APS is often associated with autoimmune events, primarily Systemic Lupus Erythematosus [2,3].

One of the severe manifestations in patients with APS is thrombotic events within the central nervous system, typically involving arterial thrombosis. Associated events such as strokes or transient cerebral ischemia are reported in 15 to 19.8% of cases. Additionally, symptoms like headaches, neurocognitive impairment, epilepsy, and less common movement disorders such as chorea have been described [2-4]. Extensive cohorts have reported serious neurological manifestations, with an incidence of 34% in China and 35% in Israel, respectively [5,6]. It has been determined that in patients who experience strokes in the context of APS, there are other risk factors to consider, such as age, female gender, smoking habits, hypertension, and dyslipidemia, among others [5-7]. Other neurological manifestations can also occur, such as cerebral venous thrombosis (seven per 1000 cases), which is more common in women than in men. Additionally, there are other coexisting risk factors in APS [7-9].

We present the case of a 43-year-old woman with undifferentiated connective tissue disease, who came to the emergency department due to 30 minutes of dysarthria, incoherent speech, and left leg weakness. The examination showed hypertension, mild dysarthria, and aphasia, she had no weakness; non-contrast tomography of the brain revealed no abnormality so a treatment with Alteplase was made successfully. The next day a magnetic resonance imaging of the brain showed acute cortical infarctions in the upper left parietal lobe and the posterior aspect of the left insula, confirming the diagnosis of an ischemic stroke. The objective of this report is to describe a case of a patient with APS who experienced a stroke and underwent successful thrombolysis with complete neurological recovery.

## Case presentation

This is a female patient, 43 years old, currently under the care of rheumatology due to undifferentiated connective tissue disease, with no other known medical history (at the time of admission without serological diagnosis of antibodies). She was admitted to a high-complexity hospital. The patient reported a clinical episode that began 30 minutes before her consultation, characterized by incoherent and dysarthric speech, as well as deviation of the corner of the mouth.

Additionally, she experienced a temporary decrease in strength in her left lower limb but fully recovered her strength in that extremity. A similar episode with self-limiting symptoms occurred a month before her consultation, which resolved completely without medical intervention. She denied experiencing headaches and other symptoms during the review of systems. Considering her medical history, the patient had a first episode of vaso-occlusion over 10 years ago, with symptoms and signs of digital ischemia in the first finger of her right hand, which resolved without further relevant incidents.

The patient's vital signs upon admission are presented in Table 1. On examination at the admission she was alert and oriented, with mild dysarthria and aphasia, the pupils were normal, visual fields without alteration, symmetrical facial movements, strength 5/5 in all segments, mild hypoesthesia in right extremities, and flexion plantar responses.

**Table 1:** Clinical laboratory values of the patient upon admission NIHSS: National Institute of Health Stroke Scale Score

Parameter	Values found in the patient	Normal reference value
Body temperature	36.5°C	36.5°C - 37°C
Heart rate	71 beats per minute	60-100 beats per minute
Respiratory rate	18 per minute	12-20 per minute
Mean arterial pressure	152/74 mmHg	120/80 mmHg
National Institutes of Health Stroke Scale -NIHSS score	2 score (moderate aphasia-word confusion-, and mild to moderate dysarthria)	0 NIHSS score

**Figure 1:**
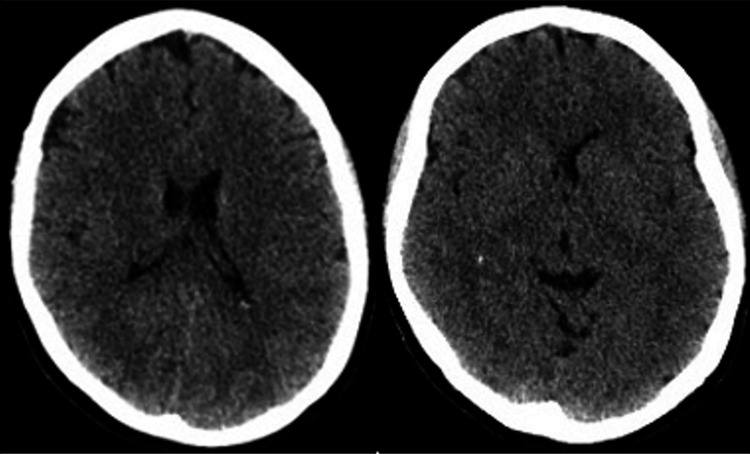
Non-contrast brain tomography of the brain The ventricular system is normal and there is no ischemic area. The Alberta Stroke Programme Early CT Score (ASPECTS) is 10/10.

**Figure 2:**
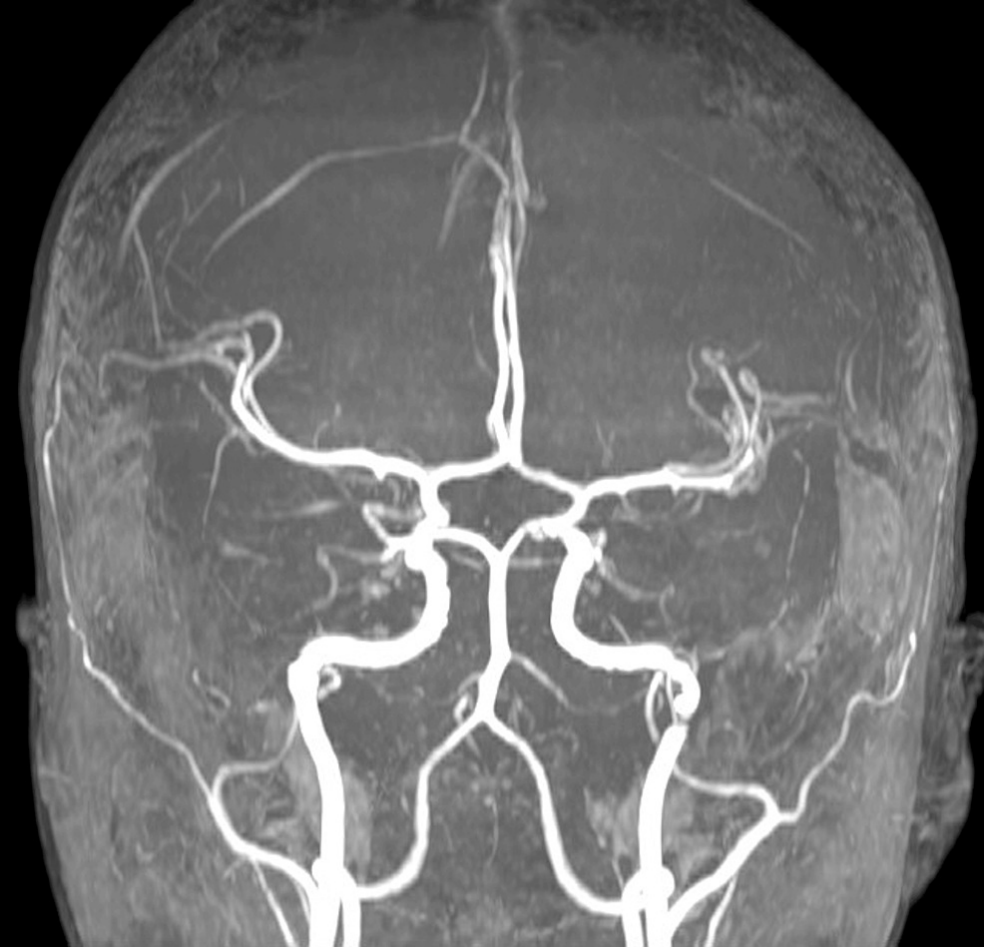
The Time-of-Flight angioresonance image The image demonstrates vascular structures of the Willis polygon with normal appearance. There are no hemodynamically significant stenoses or arteriovenous malformations.

Urgent neuroimaging was performed according to the institution's protocol, starting with a plain brain CT (Figure 1), followed by a contrast-enhanced CT angiography of the cranial vessels (Figure 2).

**Figure 3:**
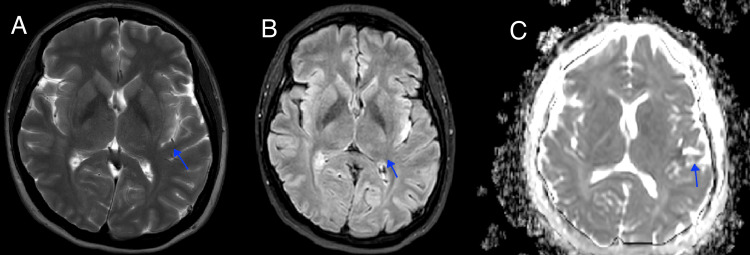
Brain cerebral magnetic resonance imaging A. T2 sequence: Hyperintensity is observed within the territory of the upper left parietal lobe. B. Fluid-attenuated inversion recovery (FLAIR) sequence: Scattered areas of restricted cortical tissue diffusion are observed with a corresponding representation in FLAIR. C. DWI-ADC: Representation in the DWI sequence, affecting the upper left parietal lobe and the posterior aspect of the insula.

This ruled out proximal vessel occlusion and intraparenchymal bleeding. Due to a high suspicion of a stroke, and in the absence of contraindications, intravenous thrombolysis with alteplase was initiated at a total dose of 54 mg, given within an hour of symptom onset. The patient showed neurological improvement before completing the medication infusion, with a National Institute of Health Stroke Scale Score (NIHSS) score of 0 at the end of the infusion. The following day, additional clinical studies were conducted to determine the etiology. A cerebral magnetic resonance imaging (Figure 3) revealed acute cortical infarctions in the upper left parietal lobe and the posterior aspect of the left insula, confirming the diagnosis of an ischemic stroke.

Results of other laboratory tests performed are presented in Table 2. Moreover, Transesophageal echocardiography showed no thrombi, valvulopathy, or abnormal interatrial communication. Moreover, there were no contraindications for thrombolysis.

**Table 2:** Additional laboratory tests of the patient after admission

Parameter	Value found in the patient	Normal reference value
Platelets	144.000 uIU/mL	150.000 to 450.000 uIU/mL
International normalized ratio (INR)	0.94	1
Hemoglobin A1c	5.2%	5.7%
High-density lipoprotein (HDL) cholesterol	52 mg/dL	60 mg/dL
Low-density lipoprotein (LDL) cholesterol	110 mg/dL	100 mg/dL
Total cholesterol	182 mg/dL	200 mg/dL
Triglycerides	99 mg/dL	150 mg/dL
Creatinine	0.72 mg/dL	0.5-1.1 mg/dL
Venereal Disease Research Laboratory (VDRL)	non-reactive	------
Thyroid-Stimulating Hormone (TSH)	8.3 uIU/mL	0.4-4.0 uIU/mL

Further autoimmunity studies were conducted. The APS profile was repeated, confirming the diagnosis (Table 3). Other studies, such as extractable nuclear antibodies (ENAs), antinuclear antibodies (ANAs), anti-neutrophil cytoplasmic antibodies (ANCAs), and a sickle cell cycling test, were negative.

**Table 3:** Autoimmunity studies Ig: Immunoglobulin

Parameter	Value found in the patient	Normal reference value
Anti-Beta-2 Glycoprotein-1 (anti-B2GP1) IgG	21 UA/ml	<5 UA/ml
anti-B2GP1 IgM	10 UA/ml	<5 UA/ml
Lupus anticoagulant diluted Russell Viper Venom Time (dRVT)	1.92 seconds (positive)	-------
The confirmatory lupus anticoagulant test ratio was 1.97 (positive)	1.97 (positive)	-------
Anticardiolipin IgM	23.7 MPL-U/ml (positive)	-------
IgG	90.9 GPL (positive)	------

This information confirmed both clinical and serological criteria for APS, considering the diagnostic criteria for the American College of Rheumatology/European Alliance of Associations for Rheumatology (ACR/EULAR) 2023 [10]. The patient was evaluated by neurology and rheumatology, who recommended indefinite anticoagulation with a vitamin K antagonist (warfarin 5 mg per day).

The patient was discharged on the seventh day with a diagnosis of acute stroke in the territory of the left middle cerebral artery, and her NIHSS score upon discharge was 0 points. That means that he did not have neurological sequelae upon discharge. Similarly, she did not present hemodynamic or neurological instability during his stay. Following the TOAST (The Trial of ORG-10172 in Acute Stroke Treatment) etiology classification, APS was determined to be the trigger for the thrombotic event. The patient signed the informed consent form related to hospital care and for academic purposes.

## Discussion

The diagnosis of APS is based on clinical and laboratory criteria, following the Sapporo criteria described in 1999 and updated in 2006 [4,5], which are detailed in Table 4. To establish the diagnosis, at least one clinical criterion and one laboratory criterion are required. Thrombocytopenia may be present in up to 16-53% of patients (5), typically due to platelet consumption resulting from increased clearance mediated by antibody adhesion. However, it is not a criterion that needs to be present in all cases [6,7].

**Table 4:** Diagnostic criteria based on the Sapporo criteria Ig: Immunoglobulin Reference no. [4]

Clinical criteria (one or more)	Laboratory criteria (one or more present on two occasions at least 12 weeks apart)
Vascular thrombosis: one or more confirmed episodes of arterial, venous, or small vessel thrombosis affecting any organ or tissue.	Lupus anticoagulant (LA) Anticardiolipin (ACL) IgM, IgG, or both (serum or plasma with medium to high titers) Anti-beta-2-glycoprotein-1 (B2GP) IgG, IgM, or both
Pregnancy-related complications: One or more unexplained deaths of morphologically normal fetuses after the 10th week of gestation, or one or more premature births (before the 34th week) due to eclampsia, preeclampsia, or placental insufficiency, or Three or more spontaneous abortions occurring before the 10th week.	

APS is associated with a wide range of clinical manifestations, including both thrombotic and non-thrombotic events. The Sapporo criteria are designed to classify APS cases. Other manifestations outside of these criteria can include thrombocytopenia, livedo reticularis, recurrent skin ulcers, valvulopathy, pulmonary hypertension, stroke, myelitis, chorea, and more [8,9]. Stroke in patients with APS has been described in some descriptive studies (19.8% stroke-11.1% transient ischemic attacks) [7-15]. Rosove and Brewer [14] described a series of patients with arterial and venous thrombosis and the presence of lupus anticoagulant (LA). Later, Khamasha et al. described 147 cases of patients with thrombotic events (54% venous, 46% arterial), finding elevated LA levels in most cases [15].

The above findings have led to the need for early anticoagulation in patients with APS who experience a thrombotic event. It has been found that the risk of recurrent events is higher in patients who do not receive anticoagulation compared to those who only receive aspirin, with a higher risk in the first 6 months after discontinuing anticoagulation [16].

It is established that APS is a factor that increases the risk of stroke, but its magnitude is still controversial and the series that have been reported on APS and stroke include patients with neurological complications following other vascular manifestations such as venous sinus thrombosis, or related stroke with valvular lesions or patent foramen ovale [15-17].

In individuals under 50 years old, 17% of strokes and 12% of transient ischemic attacks are associated with the presence of APL, making APS a leading cause of neurovascular syndrome in young patients [17]. Thrombosis is believed to be the most common pathophysiological mechanism, with large intracerebral vessels being the most affected, particularly the middle cerebral artery. Other types of embolisms have been described, including those caused by valvulopathy, carotid artery occlusion, vasculitis, and carotid and vertebral artery dissection [18]. Stroke subtypes in the context of APS can be classified as thrombotic or cardioembolic. Saidi et al. [19] described that the presence of LA is associated with cardioembolic strokes, while the presence of LA is associated with thrombotic or cardioembolic strokes.

Other neurological manifestations in the context of APS can include cerebral venous thrombosis, with an estimated 80% of cases associated with APS being the initial presentation [18-20]. Cognitive impairment is often a long-term condition, affecting 11-60% of APS cases. Other manifestations found in these patients include Posterior Reversible Encephalopathy Syndrome [17-20].

The diagnosis of APS requires, in addition to clinical criteria, the presence of laboratory criteria. Ideally, the persistence of antibody positivity should be demonstrated, as these antibodies can elevate due to viral and bacterial infections (Hepatitis C non-reactive - Hepatitis B non-reactive - HIV non-reactive - VDRL Syphilis non-reactive), among others [18]. In this case, no further demonstration was required because the patient had undergone rheumatological studies in previous years, which indicated a presumptive diagnosis of APS. However, at the time of her hospitalization, there were no reports of these paraclinical tests. In the case of stroke, the most affected territory is the middle cerebral artery. Neuroimaging typically shows cortical or lacunar cerebral infarcts, cortical atrophy, and cerebral microhemorrhages [20]. Patients with APS associated with stroke should be considered at high risk for recurrent events, and anticoagulation and antiplatelet therapy are recommended [17-20].

Primary thromboprophylaxis in APS patients with a positive lupus anticoagulant (LA) is usually done with aspirin, which has been shown to reduce the risk of the first thrombotic event by half. The standard treatment for APS patients with venous thrombosis is vitamin K antagonists (VKAs) [20]. The patient in this case presents with triple-positive APS (LA + ACL (anticardiolipin) + Beta-2 glycoprotein-B2GP), which represents a higher risk for recurrent thrombosis events (20). In such patients, anticoagulant therapy with VKAs such as Warfarin is recommended, supported by evidence from retrospective and prospective studies. There is no significant benefit of VKAs overusing aspirin (ASA) as a standalone therapy in patients with a history of stroke [17-20]. The optimal intensity of anticoagulation, indicated by an ideal INR, is not precisely defined; however, an INR in the range of 2.0 to 3.0 is generally recommended. In cases of arterial or recurrent venous thrombosis events, an INR between 3.0 and 4.0 is recommended, or combining VKA with low-dose ASA [20].

There is no concrete evidence supporting the use of direct oral anticoagulants (DOACs) over VKA therapy. DOACs are thought to target a specific coagulation factor (Xa or IIa), while VKAs inhibit the synthesis of vitamin K-dependent coagulation factors (II, VII, IX, X), potentially leading to more significant interactions with coagulation factors associated with the production of antiphospholipid antibodies. The European Alliance of Associations for Rheumatology (EULAR) in 2019 recommended a single exception for the use of rivaroxaban, which could be considered in patients with contraindications for VKAs or those unable to achieve the target INR despite adequate adherence. However, it should be avoided in patients with high levels of ACL [17-20].

In cases of Catastrophic Antiphospholipid Syndrome (CAPS), the therapy is typically more aggressive, involving a combination of glucocorticoids, heparins, plasma exchange, and intravenous immunoglobulin administration. The condition is associated with a mortality rate of 36% [15-19].

There is an association between anticoagulation and the possibility of generating false positives in the tests that are part of the APS criteria. For example, the International Society of Thrombosis and Hemostasis recommends caution in patients taking lupus anticoagulants, as the result may be a false positive or negative. In the case of this patient, at the time of stroke diagnosis, anticoagulant therapy had not been started [21].

The presence of a pre-existing diagnosis of APS is not a contraindication for thrombolysis in the case of a stroke. In this patient's case, the diagnosis of APS was presumptive upon her admission to the emergency department. Additionally, it was ruled out that she had thrombocytopenia, which would contraindicate the administration of pharmacological thrombolysis with Alteplase, leading to a successful outcome. Fibrinolytic therapy is justified in patients with thrombotic events in APS, and among these, urokinase, streptokinase, and tissue plasminogen activators can be used. Likewise, early initiation of heparin for anticoagulation during hospitalization and subsequent initiation of other anticoagulants could be considered. As a limitation of this clinical case, the unavailability of data beyond the patient's discharge weeks is noted.

## Conclusions

Cerebrovascular disease is the primary neurological manifestation in patients with APS. Therefore, emergency services personnel need to be vigilant for the clinical signs of this condition. Prompt recognition of a stroke can prevent long-term sequelae and complications when appropriate treatment is initiated. Importantly, the presence of APS as a pre-existing diagnosis is not a contraindication for thrombolysis. In cases of thrombotic manifestations associated with APS, VKAs remain the mainstay of treatment. Their prescription and monitoring should always be carefully considered. In summary, this case underscores the importance of recognizing and appropriately managing cerebrovascular events in patients with APS, which can lead to favorable outcomes when treated in a timely and effective manner.
